# Sugar-sweetened beverages increases the risk of hypertension among children and adolescence: a systematic review and dose–response meta-analysis

**DOI:** 10.1186/s12967-020-02511-9

**Published:** 2020-09-05

**Authors:** Mahdieh Abbasalizad Farhangi, Leila Nikniaz, Mahdieh Khodarahmi

**Affiliations:** 1 Drug Applied Research Center, Tabriz University of Medical Sciences, Tabriz, Iran; 2 Tabriz Health Services Management Research Center, Health Management and Safety Promotion Research Institute, Tabriz University of Medical Sciences, Tabriz, Iran; 3 Nutrition Research Center, Department of Community Nutrition, Faculty of Nutrition and Food Science, Tabriz University of Medical Sciences, Tabriz, Iran

**Keywords:** Sugar-sweetened beverages, Children, Adolescents, Blood pressure, Hypertension, SBP, DBP

## Abstract

**Background:**

In the current systematic review and meta-analysis, we summarized the studies that evaluated the effects of sugar-sweetened beverages (SSBs) intake on blood pressure among children and adolescents.

**Methods:**

In a systematic search from PubMed, Scopus, Embase and Cochrane electronic databases up to 20 April 2020, the observational studies that evaluated the association between sugar-sweetened beverages intake and hypertension, systolic or diastolic blood pressure (SBP, DBP) were retrieved.

**Results:**

A total of 14 studies with 93873 participants were included in the current meta-analysis. High SSB consumption was associated with 1.67 mmHg increase in SBP in children and adolescents (WMD: 1.67; CI 1.021–2.321; P < 0.001). The difference in DBP was not significant (WMD: 0.313; CI −0.131– 0.757; P = 0.108). High SSB consumers were 1.36 times more likely to develop hypertension compared with low SSB consumers (OR: 1.365; CI 1.145–1.626; P = 0.001). In dose–response meta-analysis, no departure from linearity was observed between SSB intake and change in SBP (P-nonlinearity = 0.707) or DBP (P-nonlinearity = 0.180).

**Conclusions:**

According to our finding, high SSB consumption increases SBP and hypertension in children and adolescents.

## Background

The increasing prevalence of obesity and weight gain in pediatric population, as a major health problem, is associated with insulin resistance, hypertension, atherogenic dyslipidemia, and pro-inflammatory state [1]. Hypertension, as one of the major component of metabolic syndrome is also associated with obesity state and has an increasing prevalence in youth [2]. Hypertension in children and adolescents is defined as average systolic blood pressure (SBP) and/or diastolic blood pressure (DBP) greater than 95th percentile for gender, age, and height on ≥ 3 occasions, while prehypertension is defined as average SBP or DBP levels that are greater than 90^th^ percentile but less than 95th percentile [3]. Hypertension and elevated blood pressure among children is associated with cardiovascular risk factors and obesity as well. Although major final outcome of CVD such as death and cardiovascular disability do not occur in hypertensive children, they are encountered with increased risk of intermediate markers of target organ damage, such as left ventricular hypertrophy, retinal vascular changes, thickening of the carotid vessel wall, and even subtle cognitive changes [4]. It is widely recognized that blood pressure levels are influenced by genetic as well as by environmental factors [5, 6]. In this regard, more than 90 different genetic polymorphisms have been identified to be associated with high blood pressure [7]. For example, a recent study reported that polymorphism of aldosterone synthase gene is linked with the development of hypertension through increasing the aldosterone level and aldosterone/renin ratio [5]. On the other hand, among environmental parameters, obesity, smoking, alcohol consumption, diet, and physical inactivity likely play a major role in development of hypertension [6].

The role of sugars in developing cardio-metabolic disorders and hypertension in children has been actively investigated. However, recently the role of sugar-sweetened beverages (SSBs) in developing hypertension particularly in children and adolescents is highlighted [8–12]. SSBs, as a liquid form of carbonated or noncarbonated energy beverages, are the principle source of added sugar in diets [13]. For instance, a cross-sectional study from China showed that SSBs provide 10–15% of total calorie intake of school students [9]. Another study in Taiwan indicated that adolescents are also one of the major groups who consume a high amount of SSBs [13]. The US Nutrition Examination Survey showed that approximately 64% of the pediatric and adolescents aged 2–19 years have daily SSB consumption contributing to 8.4% of the daily energy intake [14]. In Iran, the average SSBs intake among children and adolescents was 38.5 ± 75.0 g per day with the mean daily SSB intake of 98 ml in boys and 70 ml in girls [15].

In Australia, the average amount of 217 mL of SSB per day is consumed by youth contributing to 5.5% of their total energy intake [16]. In Mexico, SSB intake as one of the main sources of added sugar intake contributes to 8.3% of the total energy intake among children and adolescents [17]. Therefore, SSBs contain excessive amounts of energy, in the form of simple sugar. All of these figures have exceeded the recommended intake of free sugars that has been proposed by the World Health Organization to be less than 5% of total energy intakes [18]. Increased sympathetic nervous system activity [19], significant increase in blood pressure due to potential antinatriuresis effect of fructose affecting salt metabolism [9] and increased serum uric acid due to fructose metabolism [20–22] are several suggested mechanisms of the association between SSBs intake and hypertension among children and adolescents. Although numerous studies confirmed the role of high SSBs consumption in developing hypertension in youth [9, 13, 23–25], there are several inconsistencies reporting no significant association between SSB intake and blood pressure [13, 26, 27]. Moreover, childhood and adolescence are critical periods for the acquisition of healthy behaviors; therefore, the study of several indices and their co-occurrence in this ages should be a priority. In the current systematic review and meta-analysis, we aimed to summarize the studies that evaluated the association between SSBs intake and blood pressure among children and adolescents in two-class and dose–response meta-analysis.

## Materials and methods

The current study was conducted according to Preferred Reporting Items for Systematic Reviews and Meta‐Analyses (PRISMA) [28]. The completed checklist has been provided in the Additional file 1 (Additional file 1: Table S1); moreover, the abstract was written according to the 12-item PRISMA extension checklist [29].

### Data sources

A systematic search using PubMed, Scopus, Embase and Cochrane electronic databases was performed to find the studies evaluated the association between sugar-sweetened beverages intake and hypertension up to 1 April 2020. No language and time restrictions were applied. Moreover, hand-searching from reference lists of all relevant papers, previous reviews and meta-analyses was performed to cover all relevant publications. Strategy search was created using a combination of the MeSH (Medical Subject Headings) terms from the PubMed database and free text words.

### Search strategy

For the search purpose, we used MeSH (Medical Subject Heading) and non-MeSH keywords including the following: (“Child”[Mesh]) OR child[Title/Abstract]) OR childhood[Title/Abstract]) OR pediatric*[Title/Abstract]) OR adolescen*[Title/Abstract]) OR youth[Title/Abstract]) OR teenager[Title/Abstract]) OR children)) AND ((SSB[Title/Abstract]) OR Sugar-Sweetened Beverage*[Title/Abstract])) AND ((((((((“Hypertension”[Mesh]) OR hypertension[Title/Abstract]) OR HTN[Title/Abstract]) OR blood pressure [Title/Abstract]) OR systolic blood pressure[Title/Abstract]) OR diastolic blood pressure [Title/Abstract]) OR SBP [Title/Abstract]) OR DBP[Title/Abstract]) (Additional file 1: Table S2). The reviewed literatures were inserted into the EndNote software (version X8, for Windows, Thomson Reuters, Philadelphia, PA, USA). For each electronic database, search strategy was adopted.

### Study selection

In the current systematic review and meta-analysis, observational studies with the design of cross-sectional, case control or cohort evaluating the association between sugar-sweetened beverages (SSB) and hypertension (HTN), systolic blood pressure (SBP) and diastolic blood pressure (DBP) were included. The studies were included if they were (a) observational studies (b) original research as publication type; (c) reported SSB (sodas/soft drinks, carbonated beverages, non-100% fruit juice drinks, syrup-based drinks, flavored water with sugar, sports and energy drinks, chocolate milk, yogurt drinks, lemonades, Coca-Cola, Sprite, orange juice, Nutrition Express, and Red Bull and sweetened teas) intake as exposure and HTN, SBP and DBP as outcome variable; and (d) studies conducted in children and adolescents (less than 19 years of age) (e) if they reported the mean ± standard deviation (SD) of SBP or DBP or the odds ratio (OR) of HTN in subjects of the highest versus lowest SSBs category. Since there is no official definition for SSBs, they were defined as any type of above- mentioned drinks. Initially, retrieved citations were merged, duplications were eliminated and the review process was facilitated. Accordingly, the titles and abstracts of all articles were evaluated independently by 2 reviewers (MAF, LN). Full-texts of relevant articles were retrieved if meeting the eligibility criteria, and then were re-evaluated. Any disagreements were discussed and resolved by consensus.

### Risk of bias and quality assessment

The quality of cross-sectional studies was assessed by Agency for Healthcare Research and Quality (AHRQ) checklist [30]. There was no quality criteria for inclusion of the studies in the current meta-analysis. The items were scored “1” if the answer was “YES,” and “0” if the answer was “NO” or “UNCLEAR.” The final quality assessments scores were as follows: low quality = 0–3; moderate quality = 4–7; high quality ≥ 8. The details of the studies’ quality assessment are presented in Additional file 1: Table S3.

### Data collection and extraction

Data were collected according to a standard data extraction form. The following information was extracted from each study: (1) authors name; (2) publication year; (3) country of study; (4) study design; (5) age range and/or mean; (6) participants’ gender; (7) number of case and controls; (8) dietary assessment tool; (9) setting; (10) type and quantity of SSB; (11) covariates used in adjustment; (12) outcome values.

### Data synthesis and analysis

#### Two class meta-analysis of the comparison of SBP and DBP between SSB categories

The comparison of SBP and DBP between highest versus lowest category of SSB was performed by measuring the unstandardized mean differences as the effect size calculated by pooled estimate of weighted mean difference (WMD) with 95% confidence interval (CI), and the fixed effects and random effects models according to level of heterogeneity. When the mean values were missed and median and range were provided, we used the method provided by Hozo et al. [31] considering the median values as best estimate of mean for sample size more than 25 and calculating SD as follows: $S^{2} \approx \left( {\frac{1}{12}\,\left( {\frac{{\left( {a - 2m + b} \right)^{2} }}{4}\, + \left( {b\text{ - }a} \right)^{2} } \right)} \right)$). When SD of the mean difference was not available from the studies, we calculated it using the following formula: SD change = square root [(SD baseline 2 + SD final 2) − (2 × 0.8 × SD baseline × SD final)] [32], SD = IQR/1.35 (symmetrical data distribution) and SD = SEM × sqrt (n), where n is number of participants, IQR is interquartile range and SEM is standard error of the mean. When the number of individuals in each category of SSB was not provided in the manuscript, we assumed that equal number of participants is enrolled in each group. When the odds of hypertension in SSB consumers versus non-consumers were provided, ORs and 95% CIs were used to estimate the combined effects. Subgroup analysis was also performed to identify possible sources of heterogeneity according to the study setting, SSB dose, and baseline values of SBP or DBP, design, health status, sample size, region, quality score of study, gender and study design. The dose of SSB intake was converted to gram of intake per day according to food agriculture organization (FAO) guidelines for converting units, denominators and expressions [33].

Cochran’s Q test and I squared test was used to identify between-study heterogeneity; I^2^ ˂ 25%, no heterogeneity; I^2^ = 25-50%, moderate heterogeneity; I^2^ > 50% large heterogeneity [34]. The heterogeneity was considered significant if either the Q statistic had *P* value < 0.1 or I^2^ > 50%. Sensitivity analysis by exclusion of one study at a time was applied to test the influence of each individual study on overall pooled estimates and heterogeneity [35]. Begg’s funnel plots was assessed to evaluate the publication bias followed by the Egger’s regression asymmetry test and Begg’s adjusted rank correlation for formal statistical assessment of funnel plot asymmetry. The data were analyzed using STATA version 13 (STATA Corp, College Station, TX, USA), and P-values less than 0.05 were considered as statistically significant.

#### Dose–response meta-analysis of the association between SSB dose and change in SBP or DBP

For dose response meta-analysis, the eligible studies had been reported the mean (SD) of continuous variable (e.g. SBP, DBP) in at least three categories. The median point in each SSB category was also identified. If medians had not been reported in the manuscript, then approximate medians were estimated, using the midpoint of the lower and upper limits. If the highest study category was open-ended, its SSB dose was calculated by assuming that the interval was the same as the closest category. The lowest categories of SSB intake was considered as the reference dose for each study. Any potential non- linear associations of SSB intake were performed by fractional polynominal modelling (polynomials) to explore the non-linear potential effects of SSB dosage (g/d) and the study- specific parameter [36].

## Results

### Flow of studies

**Fig. 1:**
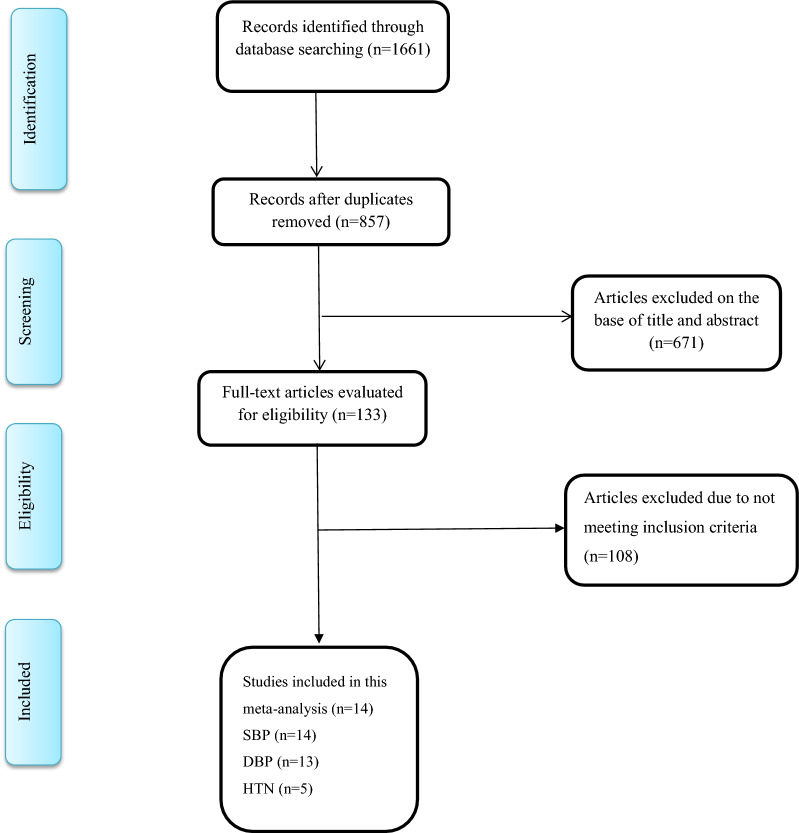
Flowchart of the literature search and study selection process

Our search strategy identified 1661 potentially relevant articles. Thereafter 857 manuscripts were remained for full text screening after removing duplicates and exclusion according to the title and abstract reading. Totally, 671 manuscripts were excluded because of their irrelevant subject, inappropriate design, being reviews including meta-analysis or systematic reviews, conferences and seminars, not relevant age groups, not evaluating the association of studied parameters. A final number of 14 manuscripts were included in the current meta-analysis (Fig. 1).

### Study characteristics

The characteristics of included studies are presented in Table 1. A total of 14 studies with 93,873 participants were included in the current meta-analysis [8–11, 13, 15, 19, 23, 24, 26, 27, 37–39]. The studies had been performed between 2009 and 2020. Totally, eleven studies reported higher SBP in higher SSB intake categories versus lower consumers [8, 10, 11, 13, 15, 19, 23, 24, 37–39]; similarly, DBP was higher among high SSB consumers compared with low consumers in six studies [10, 11, 15, 23, 24, 38]. Two studies reported no significant difference between SBP and DBP of different SSB categories [26, 27]. Four studies reported the odds of hypertension in higher SSB consumers compared with lower consumers [9, 13, 23, 24]. Different kinds of sugar-sweetened beverages were included in the self-reported SSB intake questionnaires form including: sugar sweetened sodas, carbonated beverages, caloric and sport drinks, lemonades, yogurt drinks, sweetened teas, non-100% fruit juices, cordials and other types. The age range was from 5 to 22 years old while most of the studies were performed in apparently healthy children and adolescents [8–11, 13, 15, 19, 23, 24, 27, 38, 39] and one study was performed in children with T_1_DM [26] and one in severe obesity [37]. The setting of the studies was community [8, 11, 15, 23, 39], school [9, 10, 13, 24, 27, 38], home [19] and clinic [26, 37]. The study by Chan TF et al. [13] that was conducted in two genders separately and the study by DeBoer EC et al. [19] that was performed in two age groups (5–6 years and 11–12 years) were included as two separate studies. The geographical locations of the studies were Australia [8], USA [19, 26, 39], Iran [15], Norway [37], China [9, 11, 23, 24], Brazil [10], Taiwan [13, 38] and Malaysia [27]. Almost all of the studies were cross sectional [9–11, 13, 23, 24, 26, 27, 37–39] and in three cohort studies the cross-sectional baseline data was used [8, 15, 19].

**Table 1:** The characteristics of studies included in the meta-analysis

First author	Country	Journal/year	Disease status/setting	Design/gender	Num. (total-each category)	Age range (y)	Dietary assessment tool	SSB dose (mean/median g/d)	SSB type	Main Results	Adjustments
Ambrosini GL [8]	Australia	Am J Clin Nut/2013	Apparently healthy/community	Cohort/both	1433/478	14, 17	FFQ	47.5 ± 37.1	Carbonated + cordials or and non-100% fruit juice/	SBP in higher tertiles of SSB intake was higher than lower tertile (P = 0.03). No difference in DBP was found.	Age, pubertal stage, physical fitness, dietary misreporting, maternal education, family income, BMI, healthy and Western dietary pattern scores.
Barstad LH [37]	Norway	BMC Pediatrics/2018	Severe obesity/clinic	Cross-sectional	313/(62-70)	12–18	Self-administered FFQ	None to at least 4 glasses per week ( ~ 1375)	Sugar-sweetened soda	SBP in higher intakes of SSB was higher than lower.	–
Bortsov AV [26]	USA	Acta Diabetologica/2011	Youth with T_1_DM/clinic	Cross-sectional/both	902/(304-600)	10–22	FFQ	0-750	Sugar-sweetened soda	No significant difference in SBP or DBP between three categories SSB intake	Age, sex, race/ethnicity, parental education, diabetes duration, skipping insulin, time watching TV, involvement in team sports, and total energy intake, BMI-z-score, saturated fat intake, total fiber intake
Bremer AA [39]	USA	Arch Pediatr Adolesc Med/2009	Apparently healthy/community	Cross-sectional/both	2630/(876)	12-19	FFQ	Low (≤ 20th percentile) to high (≥ 80th percentile) of the sum of the number of SSB serving equivalents	Caloric soft drinks, colas, sugar-sweetened fruit drinks, or other SSBs	Significantly higher SBP values (P = 0.03) and no difference in DBP values between low and high SSB consumers	PA, age, sex, race, energy intake (in kilocalories)
Chan TF [13]	Taiwan	Nutrients/2014	Apparently healthy/school	Cross-sectional/females	2727/(242-196)	12–16	FFQ	Non to > 750	Any type	No significant difference between SBP and DBP of different SSB categories	Age, gender, study area, PA, total calories, alcohol and smoking
Chan TF [13]	Taiwan	Nutrients/2014	Apparently healthy/school	Cross-sectional/males	2727/(406-120)	12–16	FFQ	Non to > 750	Any type	Significantly higher SBP in higher intakes compared with lower intakes of SSB (P = 0.043). No difference in DBP was observed.	Age, gender, study area, PA, total calories, alcohol and smoking.
DeBoer EC [19]	USA	Clinical Nutrition- ESPEN/2013	Apparently healthy/home	Birth cohort/both	9600	5–6	FFQ	0-750	Chocolate milk, yogurt drinks, lemonades, juices and soft drinks	Significantly higher SBP in higher versus lower SSB tertiles	Sex, height and age, ethnicity, maternal SES, BMI, PA, screen time, gestational age, birth weight, maternal BMI and paternal BMI, pubertal stage
DeBoer EC [19]	USA	Clinical Nutrition- ESPEN/2013	Apparently healthy/home	Birth cohort/both	2516/(794-905)	11-12	FFQ	0-950	Yogurt drinks, soft drinks, juices, lemonades, sport drinks and energy drinks	Significantly higher SBP in higher versus lower SSB tertiles	Sex, height and age, ethnicity, maternal SES, BMI, PA, screen time, gestational age, birth weight, maternal BMI and paternal BMI, pubertal stage
Gui ZH [23]	China	Nutrients/2017	Apparently healthy/community	Cross-sectional/both	53,151/(15 763- 17773)	6-17	FFQ	0-500	Coca-Cola, Sprite, orange juice, Nutrition Express, and Red Bull	Significantly higher SBP and DBP in higher versus lower SSB intakes (P < 0.001), no difference in odds of HTN in different SSB intakes.	Age, sex, and residence, maternal education, paternal education, family income, screen time, and PA, meat and fried food for overweight, obesity, and abdominal obesity; and meat, fried food, height, and BMI for blood pressure.
He B [24]	China	J AtherosclThromb/ 2018	Apparently healthy/school	Cross-sectional/both	2032/(440-705)	6–18	FFQ	0-120	Carbonated drinks, juices, and sports and sweet tea beverages.	Significantly higher SBP and DBP in higher versus lower SSB intakes (P < 0.001).	Age, gender, physical activities, sleeping duration, sedentary behavior, and dietary information
Lin WT [38]	Taiwan	Int J Obesity/2013	Apparently healthy/School	Cross-sectional/both	2727/(164-317)	12-16	FFQ	non-intake to ≥ 1000 ml/d	SSB, including soft drinks, fruit drinks and sweetened teas.	Significant increase in SBP (3.47 mmHg; P = 0.004) and no significant change in DBP (p = 0.514) in higher versus lower SSB consumers.	The study area, age, gender, PA, total calories, the intake of meat, seafood, fruit, fried food and a food with jelly/honey, as well as for alcohol drinking and cigarette smoking.
Loh DA [27]	Malaysia	Pediatric Obes/2015	Apparently healthy/School	Cross-sectional/both	881/(293)	13	FFQ	338.75	Carbonate beverages	No significant difference in SBP and DBP between SSB tertiels.	–
Mirmiran et al. [15]	Iran	Nutr Metab/2015	Apparently healthy/Community	Cohort/both	424/(106)	6–18	FFQ	132.7	Sugar sweetened carbonated soft drinks (SSSDs) and fruit juice drinks (non-100% fruit juices)	Significantly higher SBP in highest versus lowest SSB category (P = 0.021). No difference in DBP between SSB quartiles (P = 0.52). Higher odds of HTN in highest versus lowest SSB category (2.90 (0.91–9.26); P = 0.043)	Age, sex, total energy intake, PA, family history of diabetes dietary fiber, tea and coffee, red and processed meat, fruit, and vegetable, BMI
Qin Z [9]	China	J Hyper/2018	Apparently healthy/School	Cross-sectional/both	10091/(249-203)	Grade 4: 9.04 ± 0.38 Grade 7: 12.03 ± 0.41	FFQ	Consumers/non-consumers	Sprite and Coca-Cola	Higher odds of HTN in SSB consumers versus non-consumers [OR:1.40 (1.15,1.70)]	School, parental educational attainment, PA, diet intake of meat and snacks
Souza BSN [10]	Brazil	J Hypert/2016	Apparently healthy/school	Cross-sectional/both	488/(419-25)	9–16	FFQ	500	Soft drinks, fruit drinks and sweetened teas	Significantly higher SBP and DBP in SSB consumers than non-consumers (P < 0.05)	Age, sex, BMI, PA, addition of salt to food at the table, and education of the head of the family
Zhu Z [11]	China	Pediatric Obes/2020	Apparently healthy/Community	Cross-sectional/both	3958/(343-2582)	6-17	FFQ	201.7	Nonalcoholic beverages sweetened by sugar, excluding fresh juice.	Significantly higher SBP and DBP in high consumers versus low consumers (P < 0.001; P = 0.004)	Age, gender, energy intake, pubertal stage, daily sedentary time, maternal education, household income,

*SSB* sugar sweetened beverages, *SBP* systolic blood pressure, *DBP* diastolic blood pressure, *BMI* body mass index, *FFQ* food frequency questionnaire, *HTN* hypertension, *PA* physical activity, *T*_*1*_*DM* type one diabetes mellitus

### Findings from the two-class meta-analysis of the comparison of SBP and DBP between different SSB categories

**Fig. 2:**
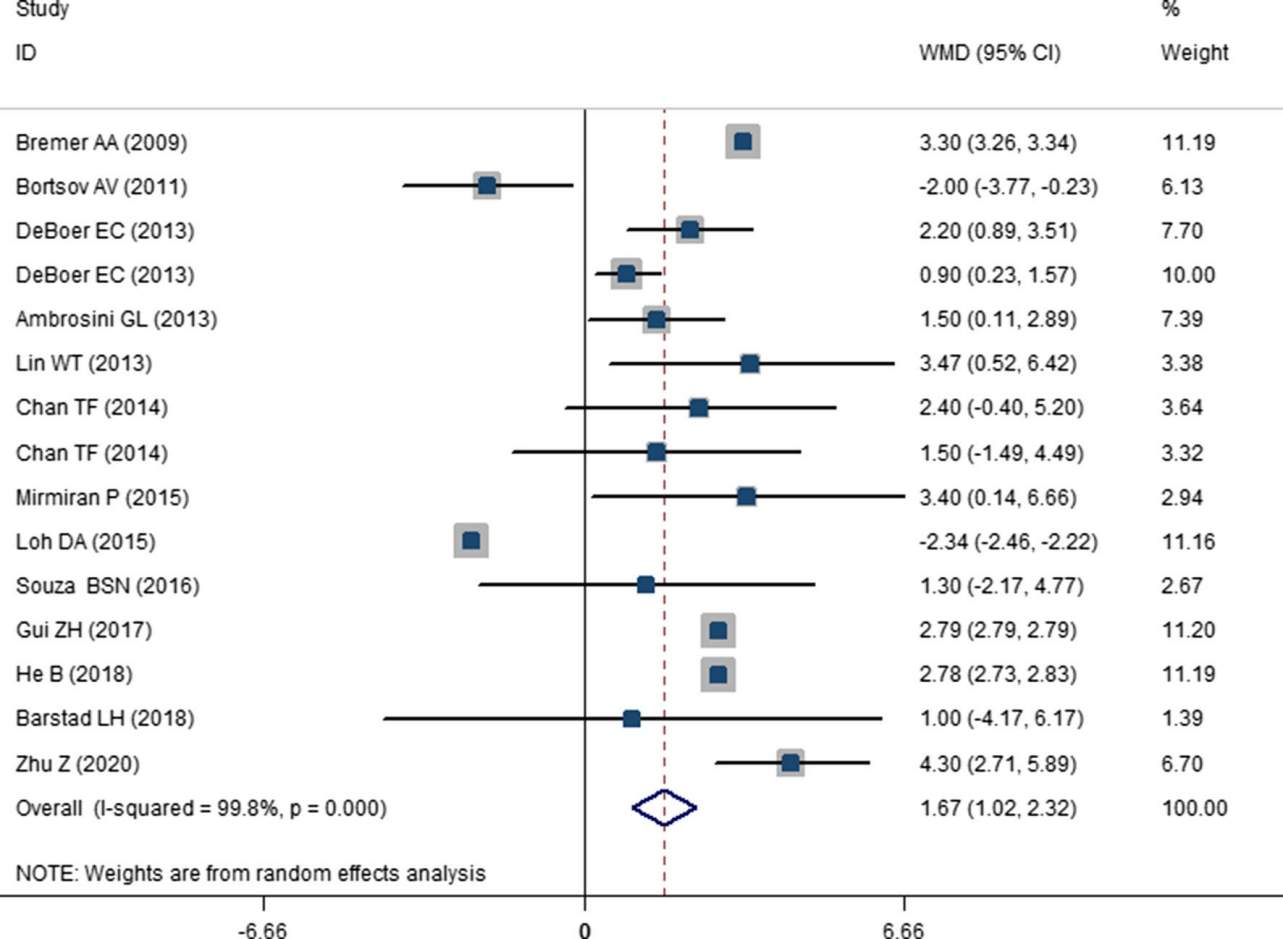
The forest plot showing the weighted mean difference (WMD) of the effect of SSBs intake on systolic blood pressure (SBP)

**Fig. 3:**
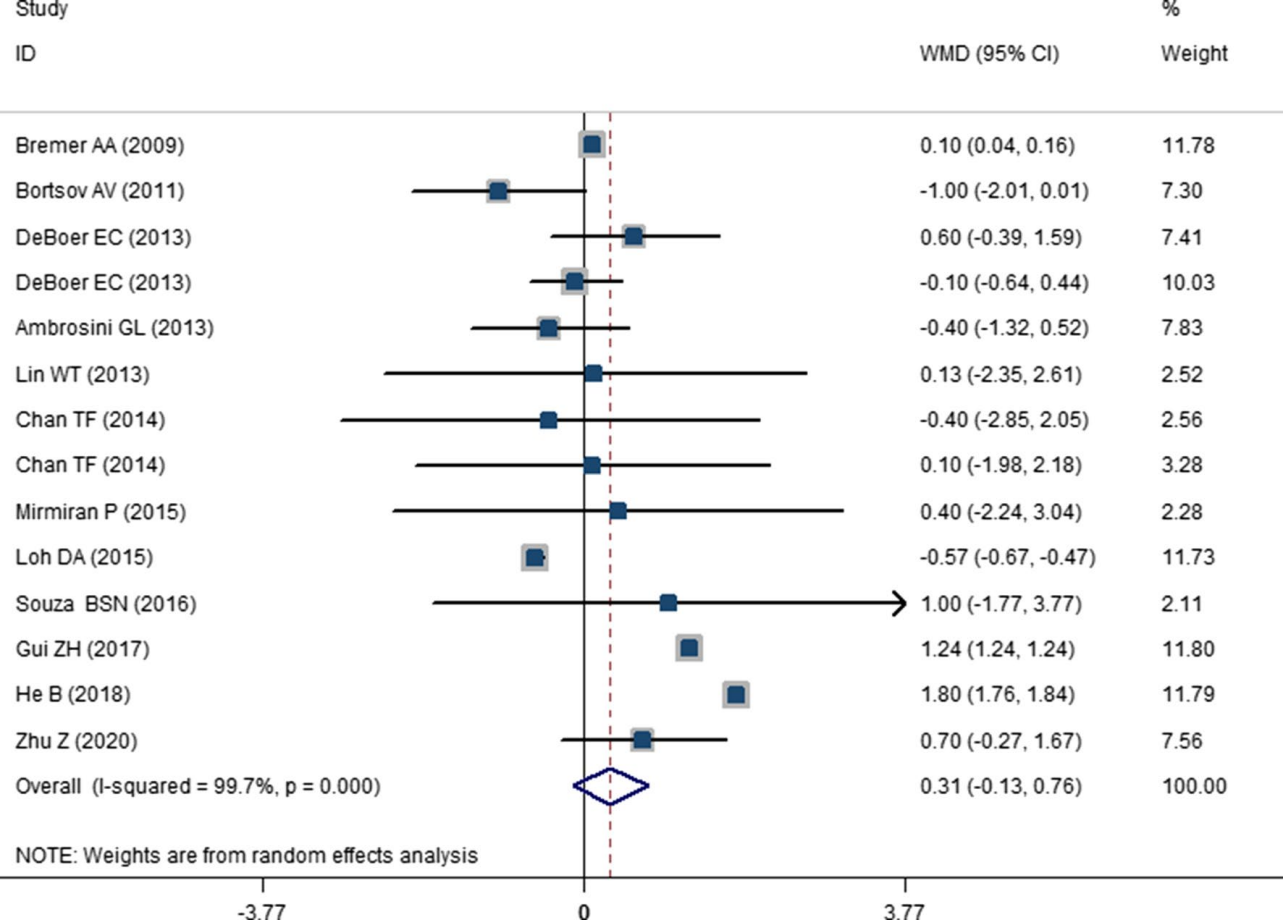
The forest plot showing the weighted mean difference (WMD) of the effect of SSBs intake on diastolic blood pressure (DBP)

**Fig. 4:**
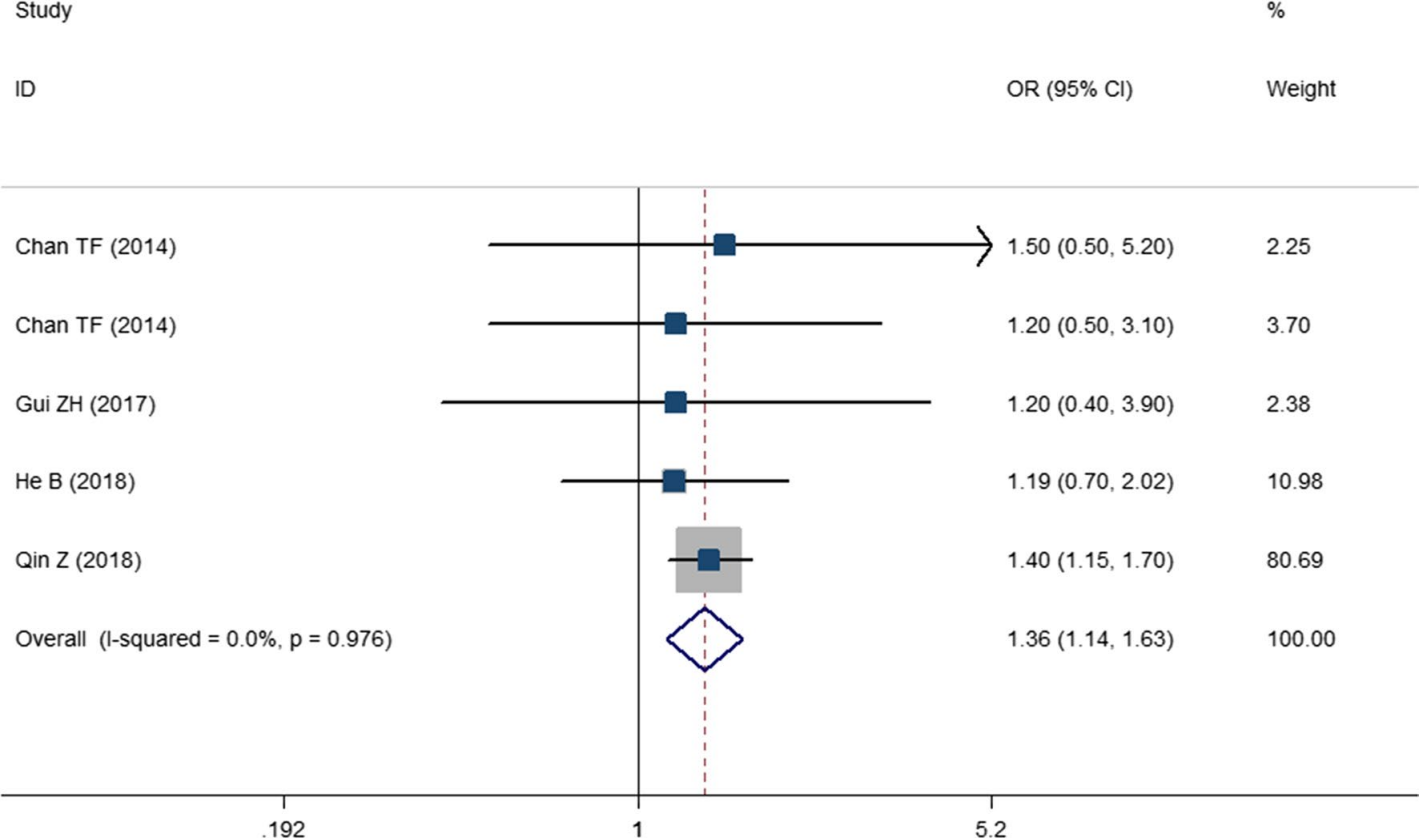
The forest plot showing the odds ratio (OR) of the association between SSBs intake and hypertension (HTN)

The results of the comparison of SBP and DBP between highest versus lowest SSB consumption categories have been presented in Figs. 2 and 3. As presented, high SSB consumption was associated with 1.67 mmHg increase in SBP in children and adolescents (WMD: 1.67; CI 1.021–2.321; P < 0.001). While, the change in DBP was not significant (WMD: 0.313; CI −0.131, 0.757; P = 0.108). Odds of hypertension in highest versus lowest SSB consumers has been shown in Fig. 4. High SSB consumers were 1.36 times more likely to develop hypertension compared with low SSB consumers (OR: 1.365; CI 1.145–1.626; P = 0.001). A significant between study heterogeneity was observed for studies that had evaluated SBP (I^2^ = 99.8; P < 0.001) and for DBP (I^2^ = 99.4; P < 0.001). However, there was no heterogeneity for the studies that had evaluated the odds of hypertension (I^2^ = 0.0; P = 0.976). For finding the source of heterogeneity, we performed subgroup analysis and the results are shown in Additional file 1: . Tables S4 and S5. As shown in these tables, subgrouping according to setting, baseline value of SBP, health status, region, gender and study quality reduced the heterogeneity for studies that evaluated the SBP values. While for DBP, the SSB dosage, setting, region, sample size, baseline DBP values, study quality, gender and design reduced the heterogeneity. Moreover, Subgroup analyses showed that a higher SSB consumption lead to a higher SBP among children and adolescents with baseline SBP greater than 110 mmHg (WMD: 0.743; CI 1.330–4.157; P < 0.001). Additionally, SSB intake might increase SBP in the studies with a sample size > 2000 (WMD: 2.720; CI 2.581–2.859; P < 0.001), school based studies (WMD: 2.780; CI 2.727–2.832; P < 0.001). Higher SSB intake also resulted in greater increase in SBP among apparently healthy subjects (WMD: WMD: 1.848; CI 0.888–2.808; P < 0.001). Accordingly, the subgrouping revealed that the high SSB intake is associated with high DBP in school based studies (WMD: 1.76; CI 1.431–2.089; P < 0.001), studies with high baseline DBP values (WMD: 0.494; CI 0.001–0.987; P = 0.049), performed in apparently healthy children or adolescents (WMD: 0.476; CI 0.023– 0.929; P = 0.039), studies with sample size greater than 2000 (WMD: 0.957; CI 0.531–1.384; P < 0.001) and studies that performed in Asia (WMD: 0.542; CI 0.024–1.060; P = 0.04).

### Finding from the dose–response meta-analysis of the association between SSB dose and blood pressure

**Fig. 5:**
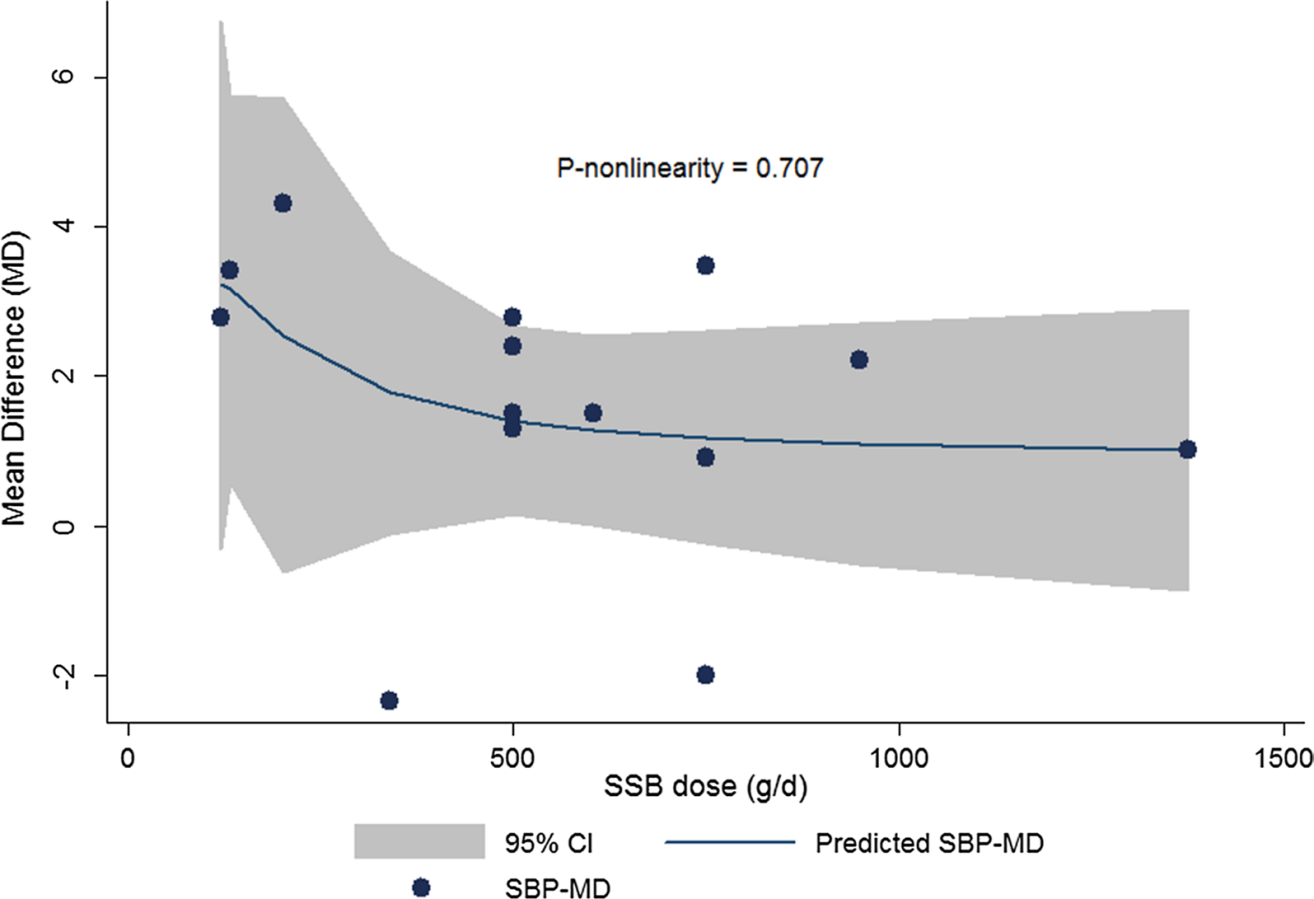
Dose– response association between the SSBs dosage and mean difference in systolic blood pressure (SBP) with the study outcomes (Linear relation (solid line) and 95% CI (gray area) of mean difference in study outcomes by 1 *g/d* increment in SSB dosage

**Fig. 6:**
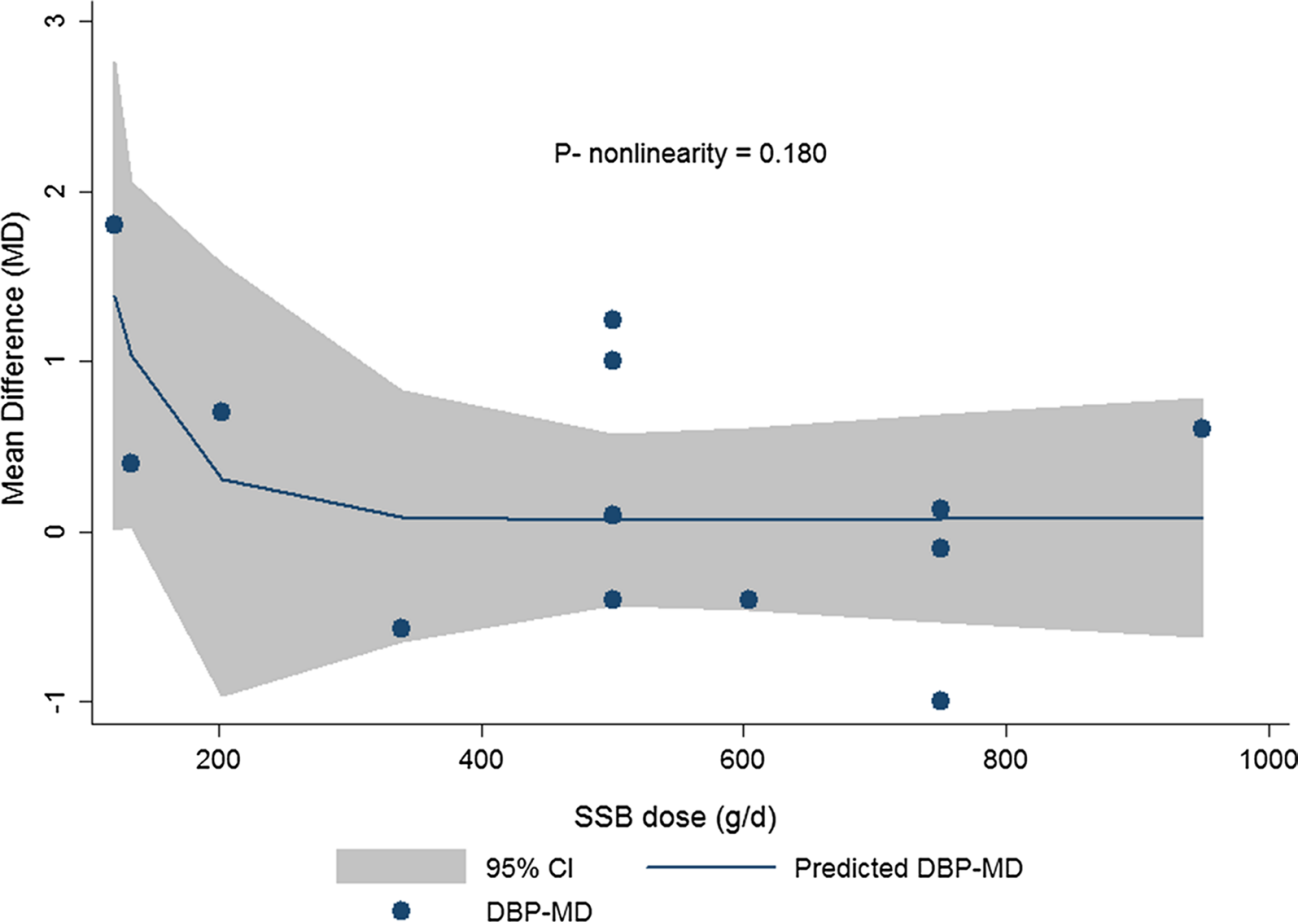
Dose– response association between the SSBs dosage and mean difference in diastolic blood pressure (DBP) with the study outcomes (Linear relation (solid line) and 95% CI (gray area) of mean difference in study outcomes by 1 *g/d* increment in SSB dosage

The details of dose–response meta-analysis are shown in Table 2 and the results for the SBP and DBP are presented in Figs. 5 and 6, respectively. According to the results of dose–response meta-analysis, no evidence of departure from linearity was observed for the association between dose of SSB with mean change in SBP (P-nonlinearity = 0.707) or DBP (P-nonlinearity = 0.180).

**Table 2:** Details of non-linear association between SSB intake, SBP and DBP

SBP _Mean difference_	Coefficient	Standard error	T	P > |t|	95% Conf. Interval
Dose_1	0.168	0.3513	0.48	0.64	−0.605– 0.941
Dose_2	0.0635	0.164575	0.39	0.707	−0.298 0.425
_cons	1.314	0.5642	2.33	0.040	0.072 2.556
DBP _Mean difference_	Coefficient	Standard error	t	P > |t|	95% Conf. Interval
Dose_1	−6.987	7.066	−0.99	0.346	−22.73–8.757
Dose_2	47.35816	32.84361	1.44	0.180	−25.82 –120.5
_cons	63.28868	1.101714	57.45	0.000	60.833– 65.743

*SSB* sugar sweetened beverages, *SBP* systolic blood pressure, *DBP* diastolic blood pressure

### Publication bias

The funnel plots are presented in Additional file 1: Figure S1a, b, c. No evidence of publication bias was observed neither for the meta-analysis of the comparison of SBP or DBP in highest versus lowest SSB categories according to Begg’s and Egger’s meta-bias tests [SBP: Begg test (*P *= 0.547) and Egger test (*P *= 0.267); DBP: Begg test (*P *= 0.115) and Egger test (*P *= 0.592)], nor for the meta-analysis of the association of hypertension with SSB intake [e.g. Begg test (*P *= 0.327) and Egger test (*P *= 0.127)].

## Discussion

According to our finding, high SSBs intake among children and adolescents was associated with higher SBP and odds of hypertension. Moreover, no evidence of departure from linearity was observed in the dose–response meta-analysis of change in SBP or DBP according to SSB dosage. A total of 14 studies with 93,873 participants were included in the current meta-analysis.

SSBs such as sugar sodas and juices are one of the main sources of excess sugar consumption containing 22 to 39 g of sugar per serving [40, 41]. The American Academy of Pediatrics (AAP) has recommended that young children refrain from intake of SSB because of its potential adverse effects on obesity and related disorders [42]. According to the last update of the clinical practice guideline which is issued by AAP, the prevalence of pediatric prehypertension and hypertension has increased to 14.8% and 16.3%, respectively [2]. In our work, high SSB intake was associated with increased systolic blood pressure and odds of hypertension; numerous trials also evaluated the effects of reduced SSB intake on blood pressure; in the study by Chen L et al. reduction in SSB intake of 1 serving/day over 18 months was associated with a 1.8 and 1.1 mmHg reduction in SBP and DBP, respectively [43]. Chiu S et al. also reported reduced systolic blood pressure after replacing sugar sweetened sodas with milk in young male adolescents [44]. Accumulating evidence has linked SSB consumption during childhood to unhealthy weight gain which itself associated with risk of health outcomes such as type 2 diabetes, metabolic syndrome, cardiovascular diseases and other obesity-related disorders in later life [45]. Therefore, intake of SSB should be limited in children and adolescents to reduce obesity-related chronic disease risk.

By using subgroup analyses, we could successfully identify possible sources of heterogeneity; such that the setting, region, gender and study quality were associated with a significant source of heterogeneity for SBP and SSB dosage, baseline DBP values, study quality, gender and design were possible source of heterogeneity across studies for DBP. Although the effect of high SSB intake on DBP was not significant, while subgrouping, the results were significant for the studies performed in apparently healthy and Asian populations, school setting, with high baseline DBP values and in large sample size studies. So, potential sources of bias were detected with the help of subgroup analyses. It seems that school is one of the best environments for children’s psychological, physical and social development [46]. Since children spend so much of their day predominantly in the school setting, the school food environment can contribute in reversing the trend towards childhood obesity [47]. Research has shown that children consume nearly 35–47% of their daily dietary intake and they are exposed to less healthful food and beverages such as SSBs and energy dense food (pizza, french fries, chips and candies) while at school [48]. It seems that improvement to the school food environment through decreasing availability of SSBs and less healthful nutritional practices can be considered as a strategy to reduce obesity and its-related complications in children and adolescents [47].

Numerous school base studies have effective strategies combating against children health problems [49–51]. WHO recommends that reduction of SSB intake among children should be implemented initially in schools by developing rules about consuming soft drinks in schools, removing vending machines selling soft drinks from school premises, provision of safe drinking water fountains in schools and other locations where children gather and promoting healthy dietary behavior in classrooms [52]. Moreover, children with higher baseline DBP values showed higher association of SSB intake with DBP; this finding showed that possibly the adverse effect of high SSB intake increased by increase in baseline blood pressure. In our research, the association between mean difference in SBP or DBP with SSB dosage did not exert a non-linear association. Therefore, increase in SBP or DBP is not a dose-dependent event after SSB consumption; this finding was also similar to the previous meta-analysis performed by Xi B et al. reporting no evidence of dose–response association between higher SSB consumption and risk of hypertension among adults (P nonlinearity = 0.82) [53]. Among the subjective dietary assessment methods such as the 24-hour dietary recall, dietary record, dietary history and food frequency questionnaire (FFQ), FFQ has been widely used in large-scale epidemiological studies [54]. It seems that the role of FFQ as a self-reported data collection tool for estimating the serving sizes might be a source of bias, this is mostly because of the difference in the FFQs structure and items and also difference in the serving definition in numerous studies. Also, in different studies outcome of study was adjusted for wide heterogenic confounders that may have affected the accuracy of dose–response estimates [53]. In the present meta-analysis, we found that SSB consumption is associated with the elevated SBP and DBP among apparently healthy subjects. However, we should take into account that the most of studies had included healthy participants in their researches and only one study performed among diabetic subjects. Therefore, the observed results may not reflect the true relationship regarding the subjects’ health status. Since the previous studies have shown that SSB intake is positively associated with diabetes and other health outcome [55], these data support the benefits of lower intake of SSBs.

Region was also another important factor affecting the SSB and DBP association. Our meta-analysis found that in the studies that performed in Asia there was a potent effect of high SSB intake on DBP, while this association was not significant for the studies that performed in USA/Oceania. Interestingly, this finding was also similar for SBP subgrouping. This finding is possibly due to this fact that most of the studies were form Asia and this high number of studies give greater power to Asian studies; also, in the previous report of global, regional, and national consumption of sugar-sweetened beverages in 187 countries, the SSB intake among Asian countries was lower than European and American countries and these findings were strongly dependent to age, country and sex of participants [56]; therefore, the role of these confounders in explaining the association between SSB intake and burden of disease should be considered. On the other hand, cultural differences among the lifestyle and socio-demographic factors play an important role in dietary intakes especially sugar; and it has been proposed as an explanation for the disparities in disease risk among ethnically diverse population [57, 58]. It seems that cultural factors by influencing on food preferences and choices may contribute to diet quality and subsequently health inequalities [57]. On the other hand, according to the latest data, childhood obesity prevalence, which coincides with the highest prevalence of hypertension and other metabolic disorders, in Latin American is among the highest in the world [59]. However, only one study from Latin American countries was included in our meta-analysis and as a result, we missed information on the relationship between SSB intake and hypertension among children and adolescents in this geographical region.

Several potential mechanisms may describe how SSB consumption could results in increasing the risk of hypertension. Hyperuricemia which is induced by a higher fructose load from sugar-sweetened beverages may leads to acute endothelial dysfunction and chronic Na retention and consequently predisposes individuals to hypertension [60, 61]. In this regard, findings from a human study showed a significant increase in blood pressure after acute administration of fructose while this effect was not seen with glucose [62]. Therefore, it has been hypothesized that the fructose in SSBs is responsible for their association with elevated blood pressure. Heredity appears to play a major role in the development of metabolic abnormalities such as hypertension especially in childhood and reports have shown heritability of childhood hypertension is estimated at 50 percent [63]. However, from included studies in our meta-analysis, only one citation [9] had included those who didn’t have a history of hypertension. On the other hand, none of included studies have adjusted for family history, thus our finding in the present meta-analysis should be interpreted with caution. Additionally, SSB consumption has been shown to be a part of an overall unhealthy dietary pattern and is correlated with unfavorable socioeconomic status [64]. There is limited research has directly compared the effect of SSB intake to other foods with regard to the risk of cardio-metabolic risk factors such as elevated blood pressure [65]. For example, Amini et al. reported western dietary pattern which contains high amount of SSB is associated with greater odds of having increased blood pressure [65]. Besides, the Dietary Approach to Stop Hypertension (DASH) which emphasizes on higher consumption of vegetables, fruits, nuts, legumes, fish, chicken, whole grains, low-fat dairy products, and lower consumption of SSBs and red meat, has been shown to be negatively associated with hypertension in adults and children [66].

Recently accumulating evidence has linked the maternal diet during pregnancy and breastfeeding to food and tastes preferences of children [67]. The fetus experiences maternal diet tastes and smells through amniotic fluids during pregnancy and afterward by breast milk [68]. Thus, maternal intake in pregnancy could program taste preference of the child towards SSB and health care providers should pay particular attention to educating women in this area.

The association between high SSBs intake and higher odds of hypertension among children and adolescents was another main finding in the present research. A large number of studies have shown that blood pressure in childhood predicts the future hypertension in adulthood [69, 70]. Hence, early interventions are warranted.

## Strength and limitations

The current systematic review and meta-analysis for the first time evaluated the dose–response association between sugar-sweetened beverage intake and hypertension in children and adolescents. Due to growing prevalence of hypertension in this population, this study has clinical and social implications regarding developing preventive strategies against high SSB consumption in children and adolescents. However, several limitations of the current meta-analysis should also be mentioned; first, using different kinds of FFQ for extraction of SSB intake is a matter of bias because this information is self-reported and has different structures and definitions between studies. Second, there were different kinds of SSBs in these studies and subgrouping according to SSB types were not possible. Moreover, different studies have reported the SSB intake with different units and these conversions might be a cause of error in estimating the accurate dosage of SSB consumption. Additionally, there were different adjustments for confounders in different studies that might affect the results.

## Conclusion

The current meta-analysis, for the first time revealed that high SSBs consumption is associated with increased SBP and odds of hypertension among children and adolescents. Although further large prospective studies and well-designed intervention studies are recommended to confirm the observed relationships, the results of the present study support recommendations to decrease the consumption of SSB to prevent and control hypertension and its complications. Developing strategic programs to reduce SSBs consumption particularly in school settings is suggested to reduce the disease burden in this population.

## Supplementary information

**Additional file 1: Supplementary Tables 1–4: PRISMA checklist, search strategies, quality assessments and subgroupings, Supplementary Figure1: Funnel plots.**

## Data Availability

The data of the current meta-analysis is available with reasonable request from the corresponding author.

## Abbreviations

SSB: Sugar-sweetened beverages
SBP: Systolic blood pressure
DBP: Diastolic blood pressure
WMD: Weighted mean difference
HTN: Hypertension
CI: Confidence interval
OR: Odds ratio
PRISMA: Preferred Reporting Items for Systematic Reviews and Meta‐Analyses

## References

[1] Bremer AA, Byrd RS, Auinger P (2010). Differences in male and female adolescents from various racial groups in the relationship between insulin resistance-associated parameters with sugar-sweetened beverage intake and physical activity levels. Clin Pediatr.

[2] Bell CS, Samuel JP, Samuels JA. Prevalence of Hypertension in Children. Hypertension (Dallas, Tex: 1979). 2019;73(1):148-52, 10.1161/hypertensionaha.118.11673. 10.1161/HYPERTENSIONAHA.118.11673 PMC6291260 30571555

[3] The fourth report on the diagnosis, evaluation, and treatment of high blood pressure in children and adolescents. Pediatrics. 2004;114(2 Suppl 4th Report):555-76, doi. 15286277

[4] Falkner B (2010). Hypertension in children and adolescents: epidemiology and natural history. Pediatric nephrology (Berlin, Germany)..

[5] Andriolo V, Dietrich S, Knüppel S, Bernigau W, Boeing H. Traditional risk factors for essential hypertension: analysis of their specific combinations in the EPIC-Potsdam cohort. Scientific Reports. 2019;9, 10.1038/s41598-019-38783-5. 10.1038/s41598-019-38783-5 PMC6365562 30728434

[6] Abdel Ghafar MT (2019). Association of aldosterone synthase CYP11B2 (-344C/T) gene polymorphism with essential hypertension and left ventricular hypertrophy in the Egyptian population. Clin Exp Hypertens.

[7] Munroe PB, Barnes MR, Caulfield MJ (2013). Advances in blood pressure genomics. Circ Res.

[8] Ambrosini GL, Oddy WH, Huang RC, Mori TA, Beilin LJ, Jebb SA (2013). Prospective associations between sugar-sweetened beverage intakes and cardiometabolic risk factors in adolescents. Am J Clin Nutr.

[9] Qin Z, Xu F, Ye Q, Zhou H, Li C, He J (2018). Sugar-sweetened beverages and school students’ hypertension in urban areas of Nanjing China. J Human Hyper.

[10] Souza Bda S, Cunha DB, Pereira RA, Sichieri R (2016). Soft drink consumption, mainly diet ones, is associated with increased blood pressure in adolescents. J Hypertens.

[11] Zhu Z, He Y, Wang Z, He X, Zang J, Guo C, et al. The associations between sugar-sweetened beverage intake and cardiometabolic risks in Chinese children and adolescents. Pediatric obesity. 2020:e12634, 10.1111/ijpo.12634. 10.1111/ijpo.12634 32196990

[12] Dehghan P, Abbasalizad Farhangi M (2020). Dietary acid load, blood pressure, fasting blood sugar and biomarkers of insulin resistance among adults: findings from an updated systematic review and meta-analysis. Int J Clin Pract.

[13] Chan TF, Lin WT, Huang HL, Lee CY, Wu PW, Chiu YW (2014). Consumption of sugar-sweetened beverages is associated with components of the metabolic syndrome in adolescents. Nutrients.

[14] Kit BK, Fakhouri TH, Park S, Nielsen SJ, Ogden CL (2013). Trends in sugar-sweetened beverage consumption among youth and adults in the United States: 1999-2010. Am J Clin Nutr.

[15] Mirmiran P, Yuzbashian E, Asghari G, Hosseinpour-Niazi S, Azizi F (2015). Consumption of sugar sweetened beverage is associated with incidence of metabolic syndrome in Tehranian children and adolescents. Nutr Metab.

[16] Brand-Miller JC, Barclay AW (2017). Declining consumption of added sugars and sugar-sweetened beverages in Australia: a challenge for obesity prevention. Am J Clin Nutr.

[17] Aburto TC, Pedraza LS, Sánchez-Pimienta TG, Batis C, Rivera JA (2016). Discretionary foods have a high contribution and fruit, vegetables, and legumes have a low contribution to the total energy intake of the Mexican Population. J Nutr.

[18] World Health Organization (WHO), Guideline: Sugars Intake for Adults and Children; World Health Organization: Geneva, Switzerland. 2015. 25905159

[19] de Boer EC, de Rooij SR, Olthof MR, Vrijkotte TGM (2018). Sugar-sweetened beverages intake is associated with blood pressure and sympathetic nervous system activation in children. Clin Nutri ESPEN.

[20] Nguyen S, Choi HK, Lustig RH, Hsu CY (2009). Sugar-sweetened beverages, serum uric acid, and blood pressure in adolescents. J Pediatr.

[21] Perng W, Tang L, Song PXK, Goran M, Tellez Rojo MM, Cantoral A, et al. Urate and Nonanoate Mark the Relationship between Sugar-Sweetened Beverage Intake and Blood Pressure in Adolescent Girls: A Metabolomics Analysis in the ELEMENT Cohort. Metabolites. 2019;9(5), 10.3390/metabo9050100. 10.3390/metabo9050100 PMC6572261 31108933

[22] Abbasalizad Farhangi M, Najafi M (2018). Dietary total antioxidant capacity (TAC) among candidates for coronary artery bypass grafting (CABG) surgery: emphasis to possible beneficial role of TAC on serum vitamin D. PLoS ONE.

[23] Gui ZH, Zhu YN, Cai L, Sun FH, Ma YH, Jing J, et al. Sugar-Sweetened Beverage Consumption and Risks of Obesity and Hypertension in Chinese Children and Adolescents: A National Cross-Sectional Analysis. Nutrients. 2017;9(12), 10.3390/nu9121302. 10.3390/nu9121302 PMC5748752 29189729

[24] He B, Long W, Li X, Yang W, Chen Y, Zhu Y (2018). Sugar-Sweetened Beverages Consumption Positively Associated with the Risks of Obesity and Hypertriglyceridemia Among Children Aged 7–18 Years in South China. J Atheroscler Thromb.

[25] Farhangi MA, Nikniaz L, Nikniaz Z, Dehghan P (2020). Dietary inflammatory index potentially increases blood pressure and markers of glucose homeostasis among adults: findings from an updated systematic review and meta-analysis. Public Health Nutr.

[26] Bortsov AV, Liese AD, Bell RA, Dabelea D, D’Agostino RB, Hamman RF (2011). Sugar-sweetened and diet beverage consumption is associated with cardiovascular risk factor profile in youth with type 1 diabetes. Acta Diabetol.

[27] Loh DA, Moy FM, Zaharan NL, Jalaludin MY, Mohamed Z (2017). Sugar-sweetened beverage intake and its associations with cardiometabolic risks among adolescents. Pediatric Obesity.

[28] Moher D, Liberati A, Tetzlaff J, Altman DG. Preferred reporting items for systematic reviews and meta-analyses: the PRISMA statement. Annals of internal medicine. 2009. 151(4):264-9, w64, 10.7326/0003-4819-151-4-200908180-00135. 10.7326/0003-4819-151-4-200908180-00135 19622511

[29] Beller EM, Glasziou PP, Altman DG, Hopewell S, Bastian H, Chalmers I (2013). PRISMA for Abstracts: reporting systematic reviews in journal and conference abstracts. PLoS Med.

[30] Quality Assessment Forms. Available from: https://www.ncbi.nlm.nih.gov/books/NBK35156/.

[31] Hozo SP, Djulbegovic B, Hozo I (2005). Estimating the mean and variance from the median, range, and the size of a sample. BMC Med Res Methodol.

[32] Wiley Online Library, J.P. Higgins, S. Green, Cochrane handbook for systematic reviews of interventions, vol. 5, 2008.

[33] Food and Agriculture Organization of the United Nations (FAO), Guidelines for converting units, denominators and expressions. 2016.

[34] Higgins JP, Thompson SG (2002). Quantifying heterogeneity in a meta-analysis. Stat Med.

[35] Shuster JJ. Cochrane handbook for systematic reviews for interventions, Version 5.1. 0, published 3/2011. Julian PT Higgins and Sally Green, Editors. Research Synthesis Methods. 2011 Jun;2(2):126-30.

[36] Fan J, Gijbels I. Local polynomial modelling and its applications: monographs on statistics and applied probability 66. CRC Press; 1996 Mar 1.

[37] Barstad LH, Júlíusson PB, Johnson LK, Hertel JK, Lekhal S, Hjelmesæth J (2018). Gender-related differences in cardiometabolic risk factors and lifestyle behaviors in treatment-seeking adolescents with severe obesity. BMC Pediatrics..

[38] Lin WT, Huang HL, Huang MC, Chan TF, Ciou SY, Lee CY, et al. Effects on uric acid, body mass index and blood pressure in adolescents of consuming beverages sweetened with high-fructose corn syrup. International journal of obesity (2005). 2013. 37(4):532-9, 10.1038/ijo.2012.121. 10.1038/ijo.2012.121 22890489

[39] Bremer AA, Auinger P, Byrd RS (2009). Relationship between insulin resistance-associated metabolic parameters and anthropometric measurements with sugar-sweetened beverage intake and physical activity levels in US adolescents: findings from the 1999-2004 National Health and Nutrition Examination Survey. Arch Pediatr Adolesc Med.

[40] Ervin RB, Kit BK, Carroll MD, Ogden CL. Consumption of added sugar among U.S. children and adolescents, 2005-2008. NCHS data brief. 2012(87):1-8, doi. 22617043

[41] Nielsen SJ, Popkin BM (2004). Changes in beverage intake between 1977 and 2001. Am J Prev Med.

[42] Heyman MB, Abrams SA. Fruit Juice in Infants, Children, and Adolescents: Current Recommendations. Pediatrics. 2017;139(6), 10.1542/peds.2017-0967.. 10.1542/peds.2017-0967 28562300

[43] Chen L, Caballero B, Mitchell DC, Loria C, Lin P-H, Champagne CM (2010). Reducing consumption of sugar-sweetened beverages is associated with reduced blood pressure: a prospective study among United States adults. Circulation.

[44] Chiu S, Siri-Tarino P, Bergeron N, Suh JH, Krauss RM. A Randomized Study of the Effect of Replacing Sugar-Sweetened Soda by Reduced Fat Milk on Cardiometabolic Health in Male Adolescent Soda Drinkers. Nutrients. 2020;12(2), 10.3390/nu12020405. 10.3390/nu12020405 PMC7071288 32033078

[45] Scharf RJ, DeBoer MD (2016). Sugar-Sweetened Beverages and Children’s Health. Annu Rev Public Health.

[46] García Bacete, F.J., G. Marande Perrin, B.H. Schneider, C. Blanchard, Effects of School on the Well-Being of Children and Adolescents, in Handbook of Child Well-Being: Theories, Methods and Policies in Global Perspective, A. Ben-Arieh, et al., Editors. 2014, Springer Netherlands: Dordrecht. p. 1251-1305.

[47] Mâsse LC, de Niet-Fitzgerald JE, Watts AW, Naylor P-J, Saewyc EM (2014). Associations between the school food environment, student consumption and body mass index of Canadian adolescents. International Journal of Behavioral Nutrition and Physical Activity..

[48] Briefel R, Wilson A, Gleason P (2009). Consumption of Low-Nutrient, Energy-Dense Foods and Beverages at School, Home, and Other Locations among School Lunch Participants and Nonparticipants. J Am Diet Assoc.

[49] Sichieri R, Paula Trotte A, de Souza RA, Veiga GV (2009). School randomised trial on prevention of excessive weight gain by discouraging students from drinking sodas. Public health nutrition..

[50] Albala C, Ebbeling CB, Cifuentes M, Lera L, Bustos N, Ludwig DS (2008). Effects of replacing the habitual consumption of sugar-sweetened beverages with milk in Chilean children. The American journal of clinical nutrition..

[51] Katan MB, de Ruyter JC, Kuijper LD, Chow CC, Hall KD, Olthof MR (2016). Impact of Masked Replacement of Sugar-Sweetened with Sugar-Free Beverages on Body Weight Increases with Initial BMI: secondary Analysis of Data from an 18 Month Double-Blind Trial in Children. PLoS ONE.

[52] Lobstein T. Reducing consumption of sugar-sweetened beverages to reduce the risk of childhood overweight and obesity. World Health Organisation. 2014 Sep.

[53] Xi B, Huang Y, Reilly KH, Li S, Zheng R, Barrio-Lopez MT (2015). Sugar-sweetened beverages and risk of hypertension and CVD: a dose-response meta-analysis. Br J Nutr.

[54] Shim JS, Oh K, Kim HC (2014). Dietary assessment methods in epidemiologic studies. Epidemiol Health.

[55] Schwingshackl L, Hoffmann G, Lampousi A-M, Knüppel S, Iqbal K, Schwedhelm C (2017). Food groups and risk of type 2 diabetes mellitus: a systematic review and meta-analysis of prospective studies. Eur J Epidemiol.

[56] Singh GM, Micha R, Khatibzadeh S, Shi P, Lim S, Andrews KG (2015). Global, Regional, and National Consumption of Sugar-Sweetened Beverages, Fruit Juices, and Milk: a Systematic Assessment of Beverage Intake in 187 Countries. PLoS ONE.

[57] Kouba J. Impact of Environment, Ethnicity, and Culture on Nutrition and Health. In: Touger-Decker R, Sirois DA, Mobley CC, editors. Nutrition and Oral Medicine. Totowa, NJ: Humana Press; 2005. p. 45-60, 10.1385/1-59259-831-5:045.

[58] Pestoni G, Krieger J-P, Sych JM, Faeh D, Rohrmann S (2019). Cultural Differences in Diet and Determinants of Diet Quality in Switzerland: results from the National Nutrition Survey menuCH. Nutrients.

[59] Caballero B, Vorkoper S, Anand N, Rivera J (2017). Preventing childhood obesity in Latin America: an agenda for regional research and strategic partnerships: childhood obesity in Latin America. Obes Rev.

[60] Cicero AFG, Fogacci F, Desideri G, Grandi E, Rizzoli E, D’Addato S (2019). Arterial Stiffness, Sugar-Sweetened Beverages and Fruits Intake in a Rural Population Sample: data from the Brisighella Heart Study. Nutrients.

[61] Caliceti C, Calabria D, Roda A, Cicero AFG. Fructose Intake, Serum Uric Acid, and Cardiometabolic Disorders: A Critical Review. Nutrients. 2017.9(4), 10.3390/nu9040395. 10.3390/nu9040395 PMC5409734 28420204

[62] Brown CM, Dulloo AG, Yepuri G, Montani JP (2008). Fructose ingestion acutely elevates blood pressure in healthy young humans. Am J Physiol Regul Integr Comp Physiol.

[63] Jung FF, Ingelfinger JR (1993). Hypertension in childhood and adolescence. Pediatr Rev.

[64] Arsenault BJ, Lamarche B, Després JP. Targeting Overconsumption of Sugar-Sweetened Beverages vs. Overall Poor Diet Quality for Cardiometabolic Diseases Risk Prevention: Place Your Bets! Nutrients. 2017.9(6), 10.3390/nu9060600. 10.3390/nu9060600 PMC5490579 28608806

[65] Amini M, Esmaillzadeh A, Shafaeizadeh S, Behrooz J, Zare M (2010). Relationship between major dietary patterns and metabolic syndrome among individuals with impaired glucose tolerance. Nutrition.

[66] Genovesi S, Orlando A, Rebora P, Giussani M, Antolini L, Nava E (2018). Effects of Lifestyle Modifications on Elevated Blood Pressure and Excess Weight in a Population of Italian Children and Adolescents. Am J Hypertens.

[67] Derks I, Sijbrands E, Wake M, Qureshi F, Ende J, Hillegers M, et al. Eating behavior and body composition across childhood: a prospective cohort study. International Journal of Behavioral Nutrition and Physical Activity. 2018;15, 10.1186/s12966-018-0725-x. 10.1186/s12966-018-0725-x PMC6167809 30285789

[68] Mennella JA, Jagnow CP, Beauchamp GK. Prenatal and postnatal flavor learning by human infants. Pediatrics. 2001;107(6):E88-E, 10.1542/peds.107.6.e88. 10.1542/peds.107.6.e88 PMC1351272 11389286

[69] Chen X, Wang Y (2008). Tracking of blood pressure from childhood to adulthood: a systematic review and meta-regression analysis. Circulation.

[70] Kalantari S, Khalili D, Asgari S, Fahimfar N, Hadaegh F, Tohidi M (2017). Predictors of early adulthood hypertension during adolescence: a population-based cohort study. BMC Public Health.

