# Supplementary material for: Sugar-sweetened beverages increases the risk of hypertension among children and adolescence: a systematic review and dose–response meta-analysis
Source: J Transl Med. 2020 Sep 5;18:344. doi: 10.1186/s12967-020-02511-9 (PMC7487688; doi:10.1186/s12967-020-02511-9)
Supplement: Supplementary file 1 — Additional file 1: Supplementary Tables 1–4: PRISMA checklist, search strategies, quality assessments and subgroupings, Supplementary Figure1: Funnel plots. [file 12967_2020_2511_MOESM1_ESM.doc]

**Title: Sugar-sweetened beverages significantly increases the risk of hypertension among children and adolescence: A systematic review and dose-response meta-analysis**

**Authors**: **Mahdieh Abbasalizad Farhangi ^1^* , Leila Nikniaz ^2^, Mahdieh Khodarahmi ^3^**

^1^ Drug Applied Research Center, Tabriz University of Medical Sciences, Tabriz-Iran.

^2^ Tabriz Health Services Management Research Center, Health Management and Safety Promotion Research Institute, Tabriz University of Medical Sciences, Tabriz-Iran

^3^ Nutrition Research Center, Department of Community Nutrition, Faculty of Nutrition and Food Science, Tabriz University of Medical Sciences, Tabriz, Iran.

***Corresponding Author**, Email address: [abbasalizadfarhangim@gmail.com](mailto:abbasalizadfarhangim@gmail.com), Tel: +98 0413 3357584

**Financial support: None**

**Conflict of interest:** The authors declare that there is no conflict of interest.

**Supplementary Material**

**Sup. Table 1.** PRISMA Checklist [1]

| **TITLE** | | |  |
| --- | --- | --- | --- |
| Title | 1 | Identify the report as a systematic review, meta-analysis, or both. | Page 1; line 1-3 |
| **ABSTRACT** | | |  |
| Structured summary | 2 | Provide a structured summary including, as applicable: background; objectives; data sources; study eligibility criteria, participants, and interventions; study appraisal and synthesis methods; results; limitations; conclusions and implications of key findings; systematic review registration number. | Page 2; lines 1-19 |
| **INTRODUCTION** | | |  |
| Rationale | 3 | Describe the rationale for the review in the context of what is already known. | Page 3 |
| Objectives | 4 | Provide an explicit statement of questions being addressed with reference to participants, interventions, comparisons, outcomes, and study design (PICOS). | Page 4; lines 6-14 |
| **METHODS** | | |  |
| Protocol and registration | 5 | Indicate if a review protocol exists, if and where it can be accessed (e.g., Web address), and, if available, provide registration information including registration number. | Page 4 lines 17-19 |
| Eligibility criteria | 6 | Specify study characteristics (e.g., PICOS, length of follow-up) and report characteristics (e.g., years considered, language, publication status) used as criteria for eligibility, giving rationale. | Page 5; lines 5-20 |
| Information sources | 7 | Describe all information sources (e.g., databases with dates of coverage, contact with study authors to identify additional studies) in the search and date last searched. | Page 4, line 17-28 |
| Search | 8 | Present full electronic search strategy for at least one database, including any limits used, such that it could be repeated. | Page 4, line 17-28 |
| Study selection | 9 | State the process for selecting studies (i.e., screening, eligibility, included in systematic review, and, if applicable, included in the meta-analysis). | Page 5; lines 5-20 |
| Data collection process | 10 | Describe method of data extraction from reports (e.g., piloted forms, independently, in duplicate) and any processes for obtaining and confirming data from investigators. | Page 5; lines 21-28  Page 6; lines 1-8 |
| Data items | 11 | List and define all variables for which data were sought (e.g., PICOS, funding sources) and any assumptions and simplifications made. | Page 5; lines 1-5 |
| Risk of bias in individual studies | 12 | Describe methods used for assessing risk of bias of individual studies (including specification of whether this was done at the study or outcome level), and how this information is to be used in any data synthesis. | Page 10- lines 1-11 |
| Summary measures | 13 | State the principal summary measures (e.g., risk ratio, difference in means). | Page 6; lines 17-28 |
| Synthesis of results | 14 | Describe the methods of handling data and combining results of studies, if done, including measures of consistency (e.g., I^2^) for each meta-analysis. | Page 6; lines 10-28 |
| Risk of bias across studies | 15 | Specify any assessment of risk of bias that may affect the cumulative evidence (e.g., publication bias, selective reporting within studies). | Page 10- lines 1-11 |
| Additional analyses | 16 | Describe methods of additional analyses (e.g., sensitivity or subgroup analyses, meta-regression), if done, indicating which were pre-specified. | Page 6; lines 10-28 |
| **RESULTS** | | |  |
| Study selection | 17 | Give numbers of studies screened, assessed for eligibility, and included in the review, with reasons for exclusions at each stage, ideally with a flow diagram. | Page 7, line 2-17 |
| Study characteristics | 18 | For each study, present characteristics for which data were extracted (e.g., study size, PICOS, follow-up period) and provide the citations. | Page 7, lines 18-28  And Table 1 |
| Risk of bias within studies | 19 | Present data on risk of bias of each study and, if available, any outcome level assessment (see item 12). | Sup. Tables 3 |
| Results of individual studies | 20 | For all outcomes considered (benefits or harms), present, for each study: (a) simple summary data for each intervention group (b) effect estimates and confidence intervals, ideally with a forest plot. | Figures 1-3  Page 8 , 9 |
| Synthesis of results | 21 | Present results of each meta-analysis done, including confidence intervals and measures of consistency. | Figures 1-3 |
| Risk of bias across studies | 22 | Present results of any assessment of risk of bias across studies (see Item 15). | Sup. Figure 1A, B, C |
| Additional analysis | 23 | Give results of additional analyses, if done (e.g., sensitivity or subgroup analyses, meta-regression [see Item 16]). | Sup. Tables 4, 5 |
| **DISCUSSION** | | |  |
| Summary of evidence | 24 | Summarize the main findings including the strength of evidence for each main outcome; consider their relevance to key groups (e.g., healthcare providers, users, and policy makers). | Page 10; lines 13-19  Page 11, 12 |
| Limitations | 25 | Discuss limitations at study and outcome level (e.g., risk of bias), and at review-level (e.g., incomplete retrieval of identified research, reporting bias). | Page 12; lines 13-20. |
| Conclusions | 26 | Provide a general interpretation of the results in the context of other evidence, and implications for future research. | Page 12, line 21-26 |
| **FUNDING** | | |  |
| Funding | 27 | Describe sources of funding for the systematic review and other support (e.g., supply of data); role of funders for the systematic review. | Page 12; line 28 |

**Sup. Table 2**. Search strategies and the number of records according to different electronic database

| **Search strategy** | **Database** | **Num. of records** |
| --- | --- | --- |
| Search (((((((((("Child"[Mesh]) OR child[Title/Abstract]) OR childhood[Title/Abstract]) OR pe?diatric*[Title/Abstract]) OR adolescen*[Title/Abstract]) OR youth[Title/Abstract]) OR teenager[Title/Abstract]) OR children)) AND ((SSB[Title/Abstract]) OR Sugar-Sweetened Beverage*[Title/Abstract])) AND (((((((("Hypertension"[Mesh]) OR hypertension[Title/Abstract]) OR HTN[Title/Abstract]) OR blood pressure[Title/Abstract]) OR systolic blood pressure[Title/Abstract]) OR diastolic blood pressure[Title/Abstract]) OR SBP[Title/Abstract]) OR DBP[Title/Abstract]) Sort by: Best Match | PubMed | 616 |
|  | Scopus | 832 |
|  | Embase | 112 |
|  | Cochrane | 101 |

**Sup. Table 3.** Agency for Healthcare Research and Quality (AHRQ) checklist to assess quality of the included studies

| **ARHQ Methodology Checklist items for Cross-Sectional study** | **Ambrosini GL [2]** | **Bortsov AV [3]** | **Bremer AA[4]** | **Chan TF [5]** | **De- Boer EC [6]** | **Gui Z [7]** | **Loh DA [8]** | **Lin WT [9]** | **Mirmiran P [10]** | **De- Moraes ACF[11]** | **Souza BSN [12]** | **Barstad LH [13]** | **He B [14]** | **Qin Z [15]** | **Zhu Z [16]** |
| --- | --- | --- | --- | --- | --- | --- | --- | --- | --- | --- | --- | --- | --- | --- | --- |
| 1) Define the source of information (survey, record review) | ⊕ | ⊕ | ⊕ | ⊕ | ⊕ | ⊕ | ⊕ | ⊕ | ⊕ | ⊕ | ⊕ | ⊕ | ⊕ | ⊕ | ⊕ |
| 2) List inclusion and exclusion criteria for exposed and unexposed subjects (cases and controls) or refer to previous publications | ⊕ | ⊕ | ⊕ | U | ⊕ | ⊕ | ⊕ | ⊕ | ⊕ | ⊕ | ⊕ | ⊕ | ⊕ | ⊕ | ⊕ |
| 3) Indicate time period used for identifying patients | ⊕ | ⊕ | ⊕ | ⊕ | ⊕ | ⊕ | ⊕ | ⊕ | ⊕ | ⊕ | ⊕ | ⊕ | ⊕ | ⊕ | ⊕ |
| 4) Indicate whether or not subjects were consecutive if not population-based | ⊕ | ⊕ | ⊕ | ⊕ | ⊕ | ⊕ | ⊕ | ⊕ | ⊕ | ⊕ | ⊕ | ⊕ | ⊕ | ⊕ | ⊕ |
| 5) Indicate if evaluators of subjective components of study were masked to other aspects of the status of the participants | U | U | U | U | U | U | U | U | U | U | U | U | U | U | U |
| 6) Describe any assessments undertaken for quality assurance purposes (e.g., test/retest of primary outcome measurements) | U | U | U | U | U | U | U | U | U | U | ⊕ | ⊕ | U | ⊕ | U |
| 7) Explain any patient exclusions from analysis | ⊕ | ⊕ | ⊕ | U | ⊕ | U | ⊕ | ⊕ | ⊕ | ⊕ | ⊕ | ⊕ | ⊕ | ⊕ | ⊕ |
| 8) Describe how confounding was assessed and/or controlled. | ⊕ | ⊕ | ⊕ | ⊕ | ⊕ | ⊕ | ⊕ | ⊕ | ⊕ | ⊕ | ⊕ | U | ⊕ | ⊕ | ⊕ |
| 9) If applicable, explain how missing data were handled in the analysis | U | ⊕ | ⊕ | U | U | U | ⊕ | ⊕ | ⊕ | ⊕ | U | ⊕ | ⊕ | ⊕ | ⊕ |
| 10) Summarize patient response rates and completeness of data collection | U | ⊕ | ⊕ | U | ⊕ | ⊕ | ⊕ | ⊕ | U | ⊕ | ⊕ | ⊕ | U | ⊕ | ⊕ |
| 11) Clarify what follow-up, if any, was expected and the percentage of patients for which incomplete data or follow-up was obtained | ⊕ | U | U | U | U | U | U | U | ⊕ | U | U | U | U | U | U |
| **Final score** | **7** | **8** | **8** | **4** | **7** | **6** | **8** | **8** | **8** | **8** | **8** | **8** | **7** | **9** | **8** |

L, low risk of bias; H, high risk of bias; U, unclear risk of bias.

**Sup. Table 4**. Results of subgroup analyses for the effects of SSB intakes on SBP values according to study or participants ‘characteristics.

| **P _between study heterogeneity_** | **I^2^, %** | **P _heterogeneity_** | **P** | **WMD (95% CI)** | **No. of studies** | **Group** |
| --- | --- | --- | --- | --- | --- | --- |
|  | 99.8 | <0.001 | 0.001 | 1.671 1.021 2.321 | 15 | **Total *** |
| <0.001 |  |  |  |  |  | **Setting** |
|  | 99.9 | < 0.001 | 0.184 | 1.881 -0.890 4.656 | 8 | Community |
|  | 0.0 | 0.636 | <0.001 | 2.780 2.727 2.832 | 3 | School |
|  | 66.6 | 0.084 | 0.026 | 1.424 0.174 2.673 | 2 | Home |
|  | 13.7 | 0.282 | 0.165 | -1.524 -3.672 0.625 | 2 | Clinic |
| <0.001 |  |  |  |  |  | **SSB dose, g/day** |
|  | 99.5 | <0.001 | <0.001 | 1.822 0.704 2.940 | 10 | ≤ 750 |
|  | 76.5 | 0.002 | 0.001 | 0.974 -0.608 2.555 | 5 | > 750 |
| 0.851 |  |  |  |  |  | **Baseline SBP mmHg** |
|  | 99.9 | <0.001 | 0.057 | 1.052 -0.031 2.136 | 9 | <110 |
|  | 46.3 | 0.114 | <0.001 | 2.743 1.330 4.157 | 6 | ≥110 |
| <0.001 |  |  |  |  |  | **Health status** |
|  | 99.8 | <0.001 | <0.001 | 1.848 0.888 2.808 | 13 | Apparently healthy |
|  | 13.7 | 0.282 | 0.165 | -1.524 -3.672 0.625 | 2 | obese, diabetes |
| <0.001 |  |  |  |  |  | **Sample size** |
|  | 92.1 | <0.001 | 0.759 | 0.370 -1.991 2.731 | 6 | ≤ 1000 |
|  | - | - | 0.035 | 1.500 0.105 2.895 | 1 | 1000-2000 |
|  | 82.8 | <0.001 | <0.001 | 2.720 2.581 2.859 | 8 | >2000 |
| <0.001 |  |  |  |  |  | **Region** |
|  | 78.8 | 0.003 | 0.459 | 0.607 -1.001 2.215 | 5 | USA |
|  | 0.0 | 0.885 | 0.033 | 1.466 0.120 2.813 | 2 | Europe/Oceania |
|  | 99.9 | <0.001 | 0.001 | 2.044 0.856 3.232 | 8 | Asia |
| <0.001 |  |  |  |  |  | **Quality score** |
|  | 93.9 | <0.001 | 0.389 | 1.206 -1.538 3.950 | 8 | 8 |
|  | 88.4 | <0.001 | <0.001 | 2.688 2.546 2.831 | 5 | 6, 7 |
|  | 0.0 | 0.667 | 0.058 | 1.980 -0.064 4.024 | 2 | > 6 |
| 0.438 |  |  |  |  |  | **Gender** |
|  | 0.0 | 0.667 | 0.058 | 1.980 -0.064 4.024 | 3 | Male/ Female |
|  | 99.8 | <0.001 | 0.002 | 1.490 0.527 2.453 | 12 | Both gender |
| <0.001 |  |  |  |  |  | **Design** |
|  | 99.9 | <0.001 | 0.007 | 1.438 0.398 2.479 | 12 | Cross-sectional |
|  | 57.8 | 0.094 | 0.009 | 1.668 0.423 2.914 | 3 | Longitudinal |

* Note that the study by Chan TF et al [5] that conducted in two genders separately and the study by DeBoer EC et al [6] in two age ranges (5-6 years and 11-12 years) are included as two separate studies.

**Sup. Table 5**. Results of subgroup analyses for the effects of SSB intakes on DBP values according to study or participants ‘characteristics.

| **P _between study heterogeneity_** | **I^2^, %** | **P _heterogeneity_** | **P** | **WMD (95% CI)** | **No. of studies** | **Group** |
| --- | --- | --- | --- | --- | --- | --- |
|  | 99.4 | <0.001 | 0.108 | 0.313 -0.131 0.757 | 14 | **Total *** |
| <0.001 |  |  |  |  |  | **Setting** |
|  | 99.5 | <0.001 | 0.740 | 0.183 -0.896 1.261 | 8 | Community |
|  | 3.1 | 0.356 | <0.001 | 1.760 1.431 2.089 | 3 | School |
|  | 31.8 | 0.226 | 0.710 | 0.121 -0.517 0.758 | 2 | Home |
|  | - | - | 0.053 | -1.000 -2.014 0.014 | 1 | Clinic |
| 0.051 |  |  |  |  |  | **SSB dose, g/day** |
|  | 99.6 | 0.000 | 0.126 | 0.367 -0.103 0.837 | 10 | ≤ 750 |
|  | 0.0 | 0.725 | 0.352 | 0.400 -0.443 1.243 | 4 | > 750 |
| <0.001 |  |  |  |  |  | **Baseline DBP mmHg** |
|  | 0.0 | 0.613 | 0.538 | -0.145 -0.605 0.316 | 4 | <60 |
|  | 99.6 | <0.001 | 0.049 | 0.494 0.001 0.987 | 10 | ≥ 60 |
| <0.001 |  |  |  |  |  | **Health status** |
|  | 99.5 | <0.001 | 0.039 | 0.476 0.023 0.929 | 13 | Apparently healthy |
|  | - | - | 0.053 | -1.000 -2.014 0.014 | 1 | Diabetes |
| <0.001 |  |  |  |  |  | **Sample size** |
|  | 48.1 | 0.103 | 0.378 | -0.284 -0.915 0.347 | 6 | ≤ 1000 |
|  | - | - | 0.394 | -0.400 -1.320 0.520 | 1 | 1000-2000 |
|  | 99.3 | <0.001 | <0.001 | 0.957 0.531 1.384 | 7 | >2000 |
| <0.001 |  |  |  |  |  | **Region** |
|  | 45.8 | 0.137 | 0.808 | -0.086 -0.776 0.605 | 5 | USA/Oceania |
|  | 99.6 | <0.001 | 0.04 | 0.542 0.024 1.060 | 9 | Asia |
| <0.001 |  |  |  |  |  | **Quality of study** |
|  | 46.2 | 0.098 | 0.471 | -0.229 -0.851 0.393 | 7 | 8 |
|  | 99.5 | <0.001 | <0.001 | 0.869 0.447 1.291 | 5 | 6, 7 |
|  | 0.0 | 0.761 | 0.893 | -0.109 -1.697 1.478 | 2 | > 6 |
| 0.096 |  |  |  |  |  | **Gender** |
|  | 0.0 | 0.761 | 0.893 | -0.109 -1.697 1.478 | 2 | Male/ Female |
|  | 99.5 | <0.001 | 0.091 | 0.387 -0.062 0.837 | 12 | Both gender |
| <0.001 |  |  |  |  |  | **Design** |
|  | 99.6 | <0.001 | 0.112 | 0.399 -0.093 0.892 | 11 | Cross-sectional |
|  | 0.0 | 0.466 | 0.766 | 0.071 -0.397 0.540 | 3 | Longitudinal |

* Note that the study by Chan TF et al [5] that conducted in two genders separately and the study by DeBoer EC et al [6] in two age ranges (5-6 years and 11-12 years) are included as two separate studies.

**(A)**

**
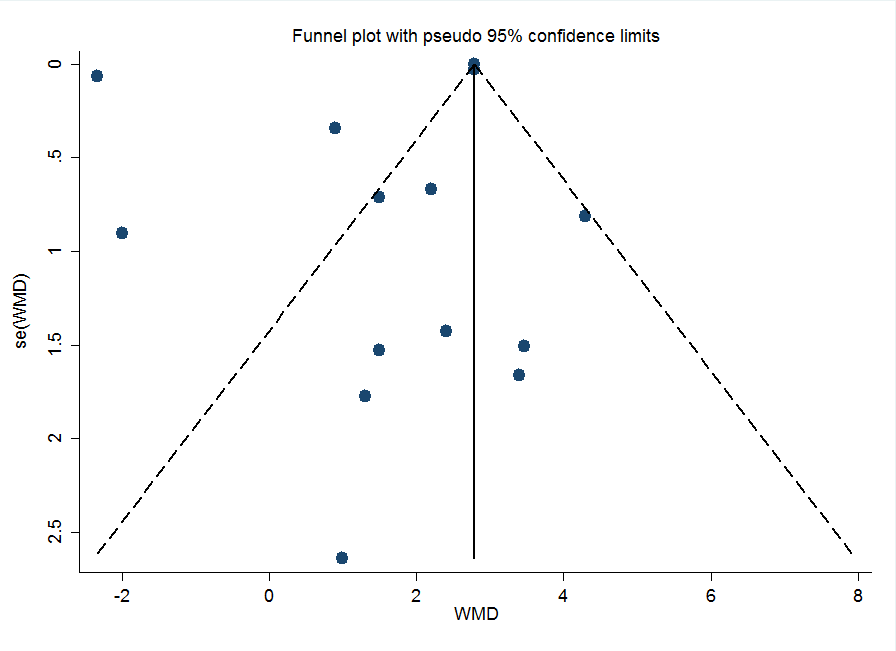
**

**(B)**

**
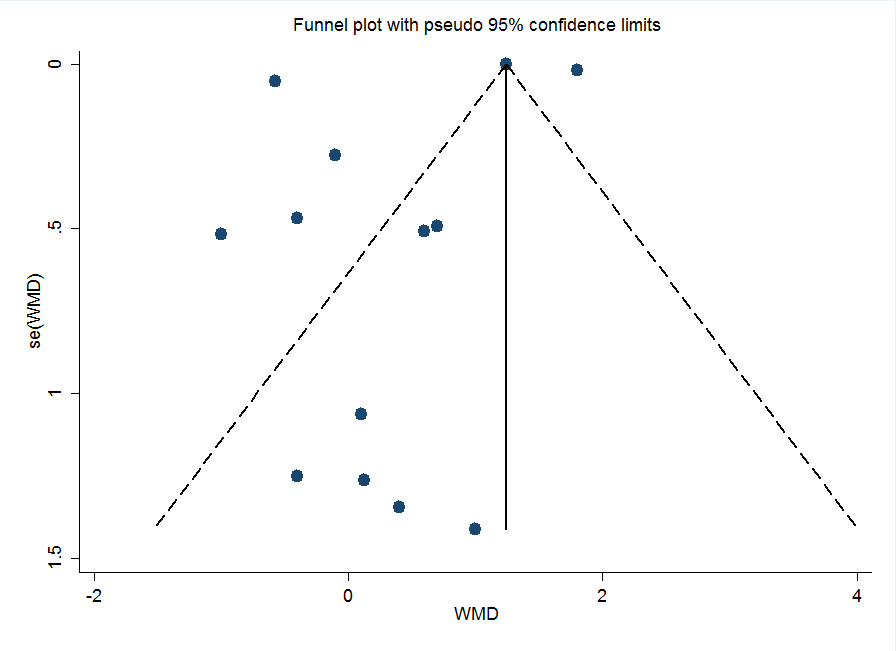
**

**(C)**

**
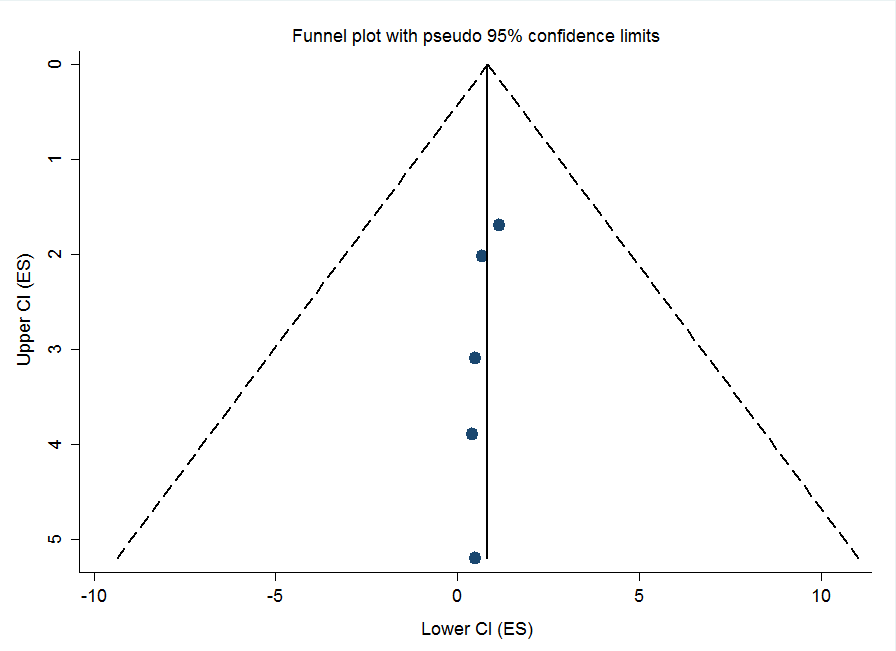
**

**Sup Figure 1.** Begg's funnel plot (with pseudo 95% CIs) of the WMD versus the se (WMD) for studies evaluating the association between SSBs intake and (A) systolic blood pressure (B) diastolic blood pressure (C) odds of hypertension

**References**

1. Moher, D., A. Liberati, J. Tetzlaff, D.G. Altman. Preferred reporting items for systematic reviews and meta-analyses: the PRISMA statement*.* Annals of internal medicine 2009; 151(4): 264-269.

2. Ambrosini, G.L., W.H. Oddy, R.C. Huang, T.A. Mori, L.J. Beilin, S.A. Jebb. Prospective associations between sugar-sweetened beverage intakes and cardiometabolic risk factors in adolescents*.* Am J Clin Nutr; 98(2): 327-34.

3. Bortsov, A.V., A.D. Liese, R.A. Bell, D. Dabelea, R.B. D'Agostino, Jr., R.F. Hamman, et al. Sugar-sweetened and diet beverage consumption is associated with cardiovascular risk factor profile in youth with type 1 diabetes*.* Acta Diabetol; 48(4): 275-282.

4. Bremer, A.A., P. Auinger, R.S. Byrd. Relationship between insulin resistance-associated metabolic parameters and anthropometric measurements with sugar-sweetened beverage intake and physical activity levels in US adolescents: findings from the 1999-2004 National Health and Nutrition Examination Survey*.* Arch Pediatr Adolesc Med; 163(4): 328-35.

5. Chan, T.F., W.T. Lin, H.L. Huang, C.Y. Lee, P.W. Wu, Y.W. Chiu, et al. Consumption of sugar-sweetened beverages is associated with components of the metabolic syndrome in adolescents*.* Nutrients; 6(5): 2088-103.

6. de Boer, E.C., S.R. de Rooij, M.R. Olthof, T.G.M. Vrijkotte. Sugar-sweetened beverages intake is associated with blood pressure and sympathetic nervous system activation in children*.* Clin Nutr ESPEN; 28: 232-235.

7. Gui, Z.H., Y.N. Zhu, L. Cai, F.H. Sun, Y.H. Ma, J. Jing, et al. Sugar-Sweetened Beverage Consumption and Risks of Obesity and Hypertension in Chinese Children and Adolescents: A National Cross-Sectional Analysis*.* Nutrients; 9(12).

8. Loh, D.A., F.M. Moy, N.L. Zaharan, M.Y. Jalaludin, Z. Mohamed. Sugar-sweetened beverage intake and its associations with cardiometabolic risks among adolescents*.* Pediatr Obes; 12(1): e1-e5.

9. Lin, W.T., H.L. Huang, M.C. Huang, T.F. Chan, S.Y. Ciou, C.Y. Lee, et al. Effects on uric acid, body mass index and blood pressure in adolescents of consuming beverages sweetened with high-fructose corn syrup*.* International Journal of Obesity 2013; 37(4): 532-539.

10. Mirmiran, P., E. Yuzbashian, G. Asghari, S. Hosseinpour-Niazi, F. Azizi. Consumption of sugar sweetened beverage is associated with incidence of metabolic syndrome in Tehranian children and adolescents*.* Nutr Metab (Lond) 2015; 12: 25.

11. de Moraes, A.C.F., J.M. Fernández-Alvira, T. Rendo-Urteaga, C. Julián-Almárcegui, L. Beghin, A. Kafatos, et al. Effects of clustering of multiple lifestyle-related behaviors on blood pressure in adolescents from two observational studies*.* Preventive Medicine 2016; 82: 111-117.

12. Souza, B.D.S.N., D.B. Cunha, R.A. Pereira, R. Sichieri. Soft drink consumption, mainly diet ones, is associated with increased blood pressure in adolescents*.* Journal of Hypertension 2016; 34(2): 221-225.

13. Barstad, L.H., P.B. Júlíusson, L.K. Johnson, J.K. Hertel, S. Lekhal, J. Hjelmesæth. Gender-related differences in cardiometabolic risk factors and lifestyle behaviors in treatment-seeking adolescents with severe obesity*.* BMC Pediatrics 2018; 18(1).

14. He, B., W. Long, X. Li, W. Yang, Y. Chen, Y. Zhu. Sugar-sweetened beverages consumption positively associated with the risks of obesity and hypertriglyceridemia among children aged 7–18 years in south China*.* Journal of Atherosclerosis and Thrombosis 2018; 25(1): 81-89.

15. Qin, Z., F. Xu, Q. Ye, H. Zhou, C. Li, J. He, et al. Sugar-sweetened beverages and school students' hypertension in urban areas of Nanjing, China /692/499 /692/700/459/284 article*.* Journal of Human Hypertension 2018; 32(6): 392-396.

16. Zhu, Z., Y. He, Z. Wang, X. He, J. Zang, C. Guo, et al. The associations between sugar-sweetened beverage intake and cardiometabolic risks in Chinese children and adolescents*.* Pediatr Obes 2020: e12634.
